# The Rapid Changes in Bodyweight and Glycemic Control Are Determined by Pre-status After Bariatric Surgery in Both Genders in Young Chinese Individuals

**DOI:** 10.7759/cureus.46603

**Published:** 2023-10-06

**Authors:** Song Wen, Min Gong, Tingfeng Wang, Mingyue Zhou, Meiyuan Dong, Yanyan Li, Chenglin Xu, Yue Yuan, Ligang Zhou

**Affiliations:** 1 Endocrinology, Shanghai Pudong Hospital, Shanghai, CHN; 2 Surgery, Shanghai Pudong Hospital, Shanghai, CHN; 3 Gynecology, University of California San Francisco, San Francisco, USA

**Keywords:** gender disparity, obesity, hba1c, bodyweight, bariatric surgery

## Abstract

Purposes: The primary aim of this clinical study is to identify the factors associated with rapid glycemic, bodyweight, and lipid profile remission in young obese patients following bariatric surgery.

Materials and methods: In a total of 131 Chinese in-patients at Shanghai Pudong Hospital, China, we retrospectively analyzed in-patient data of metabolic parameters, including BMI, waist circumference, blood pressure (BP), and blood laboratory tests, such as plasma lipids and lipoprotein, hemoglobulin A1c (HbA1c), and oral glucose tolerance tests (OGTT) before bariatric surgery. We followed up these indices at the first month, third months, half-year, and one year later.

Results: The results showed that bodyweight, BP, fasting plasma glucose (FPG), HbA1c, and triglyceride (TG) levels decreased significantly in one to three months following surgery in both male and female patients (p<0.05). We demonstrated that age (male: β=-0.181; female: β=-0.292) and the pre-operation HbA1c levels (male: β=0.935; female: β=0.919) were independent predictors of HbA1c reduction in both young obese male and female patients in three months after surgery. For body weight loss, age (β=-0.229) and pre-operation bodyweight (β=0.735) are the predictors in females, but only pre-operation body weight (β=0.798) is the independent predictor in obese young male patients.

Conclusion: This study discovered that changes in bodyweight were determined by age, pre-operation status of bodyweight, and HbA1C in obese young Chinese.

## Introduction

Obesity or overweight is the leading cause of type 2 diabetes mellitus (T2D) today [1]. Obesity (defined as a BMI of 30 kg/m^2^) was 10.8% and 15.0% in men and women, respectively, in 2014. Overweight (defined as a BMI of 25-30 kg/m^2^) was 24.4% and 27.9% in men and women, respectively. By 2025, it is expected that the global prevalence of obesity will climb to 18% in men and 21% in women [2]. Obesity is a spectrum of disorders characterized by the BMI criteria (a BMI of 30 kg/m^2^ is considered obese by the WHO in Caucasians; however, 28 kg/m^2^ is considered obese in Chinese) [3]. Despite the fact that bariatric surgery was the approach to treating morbid obesity, recent research and clinical settings have repeatedly shown that it can result in glucose metabolism remission [4,5]. Multiple studies show that bariatric surgical therapies, such as Roux-en-Y gastric bypass (RYGB), vertical sleeve gastrectomy (VSG), and biliopancreatic diversion (BPD), could improve diabetes in the majority of patients, with results that are independent of bodyweight loss [6]. The underlying mechanisms may include caloric restrictions or malabsorption, changes in gut hormones (especially glucagon-like peptide 1 (GLP-1)), gut microbiota, and others [7]. Moreover, few studies in the literature compare the metabolic consequences of bariatric surgery on male and female patients. As a result, the impacts of gender variation in glucose metabolism, bodyweight, blood pressure, lipids, and lipoprotein, as well as the predictor of rapid change of previous clinical parameters in different genders, were studied retrospectively in this study. The purpose of this study is to figure out how to tailor treatment for different genders and predict surgery outcomes.

## Materials and methods

Materials and methods

Ethics Statements

The study, including surveys, sampling, examinations, and raw data access or utilization, has obtained ethics approvals and permissions from the Ethics Committee of Shanghai Pudong Hospital (No. WZ-010). The informed consent was received from study participants before the whole study. The guidelines outlined and procedures were under the Declaration of Helsinki. All the data used in this study were anonymized before its use.

Source of In-patient Data

Patients' information was collected from the in-patient information system at Shanghai Pudong Hospital. Based on the Chinese diagnosis standard for obesity (BMI≥28 kg/m^2^), we included 131 adult, obese patients from the year 2016-2019 who underwent bariatric surgery (including 119 sleeve gastrectomy (SG), and one biliopancreatic diversion with duodenal switch (BPD-DS), three stomach-intestinal pylorus sparing (SIPS), six RYGB, and two adjustable gastric band (AGB)) in the Department of Gastrointestinal Surgery. These individuals had their pancreatic function evaluated in the Department of Endocrinology prior to surgery, including OGTT and insulin release assays. Secondary obesity, such as Cushing syndrome, is among the exclusion criteria.

Pre-operation Characteristics Evaluation

Table 1 shows the pre-operation metabolic characteristic data, including gender, age, BMI, BP, FPG, HbA1c, plasma lipids, and lipoprotein.

**Table 1:** Basic metabolic information of patients before surgery intervention, 2016-2019. Basic metabolic information of patients before surgery intervention, 2016-2019 Note: SBP: systolic blood pressure; DBP: diastolic blood pressure; WC: waist circumference Data are mean ± SD unless otherwise indicated; ***: p<0.001

Characteristic	Male	Female	P value
Numbers (n)	44	87	-
Age (years)	32.61±11.378	33.70±12.365	0.626
BMI/ male (kg/m^2^)	39.755±7.363	37.467±7.361	0.095
WC (cm)	124.840±16.775	114.720±15.616	0.001***
SBP (mmHg)	140.160±13.207	135.790±17.975	0.156
DBP (mmHg)	88.090±9.771	85.700±10.923	0.223
TG (mmol/l)	2.174±1.260	1.884±1.107	0.179
LDL (mmol/l)	3.229±1.002	3.230±0.924	0.994
HDL (mmol/l)	1.101±0.873	1.130±0.283	0.773
HbA1c (%)	7.384±1.790	7.059±1.649	0.302
FPG/male (mmol/l)	7.240±2.610	6.700±2.463	0.250
HOMA-IR	2.650±1.500	2.071±0.869	0.064

Analyses on Bodyweight, BMI, Waist Circumference, BP, FPG, and OGTT Before and After Bariatric Surgery

Bodyweight, BMI, waist circumference, BP, OGTT, insulin release tests, HbA1c, plasma lipids and lipoprotein, homeostasis model assessment of insulin resistance (HOMA-IR), homeostasis model assessment of β-cell function (HOMA-β) were analyzed before surgery. Then, we examined bodyweight, BMI, waist circumference, blood pressure, FPG, HbA1c, and plasma lipids and lipoprotein levels one month, three months, six months, and one year after surgery.

Statistical analyses

Statistics analyses were performed in Statistical Product and Service Solutions (SPSS) (version 26.0; IBM SPSS Statistics for Windows, Armonk, NY) and Prism (GraphPad, version 9.0). Two-way ANOVA was used to compare changes in the levels of bodyweight, HbA1c, FPG, and plasma lipids. Multilinear regression analyses were performed to establish the models of the proper predictors of bodyweight and HbA1c changes in three months, respectively. Statistical significance was set at p<0.05 level for all analyses.

## Results

Analyses of the glycemic state and pancreatic function prior to bariatric surgery

**Figure 1:**
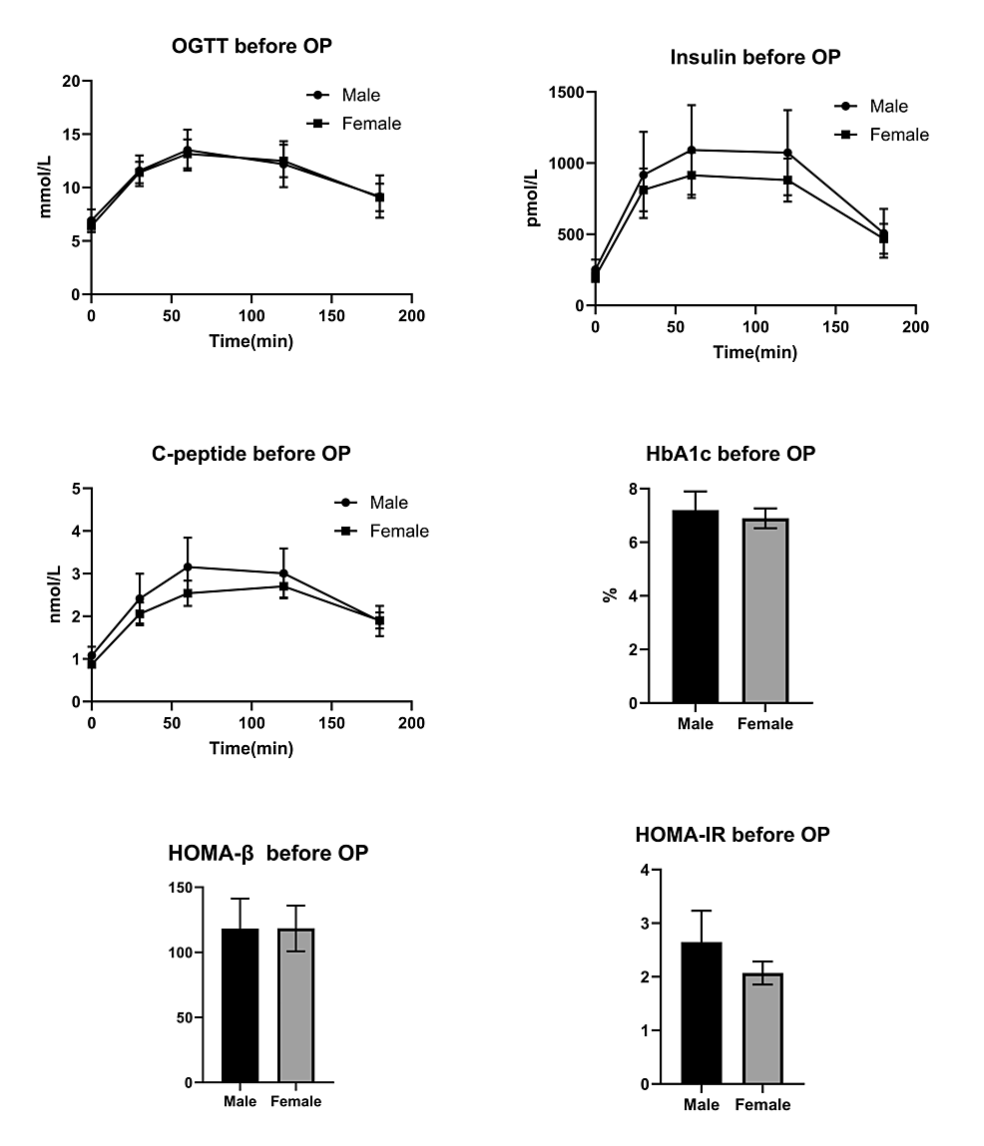
The indices related to glycemic state and pancreatic function before bariatric surgery. The figure showed the results of the analyses on OGTT (A), insulin (B), and c-peptide release tests (C), HbA1c (D), HOMA-IR (E), and HOMA-β (F). All the indices were not significantly different between the male and female patients (p>0.05). Note: OGTT: oral glucose tolerance tests; OP: bariatric surgery; HOMA-IR: homeostasis model assessment of insulin resistance; HOMA-β homeostasis model assessment of β-cell function

We first observed that male and female patients had comparable venous glucose plots evaluated by OGTT. Then, we evaluated the insulin and C-peptide release tests and found that there were no significant differences between the genders. Furthermore, our findings indicated that HbA1c, HOMA-β, and HOMA-IR were equivalent in both male and female patients (Fig. 1, Table 2).

**Table 2:** Comparison between male and female patients on the oral glucose tolerance test, insulin and C-peptide release test, HbA1c, HOMA-β, and HOMA-IR at pre-operation. Note: HbA1c: glycated hemoglobin A1c; HOMA-IR: homeostatic model assessment of insulin resistant; HOMA-β: homeostatic model assessment of β-cell function

		Males	Females	P value
Oral glucose tolerance test (OGTT)				
Glucose (mmol/L)	0 min	6.904±2.697	6.405±2.348	>0.9999
	30 min	11.574±3.709	11.413±4.036	>0.9999
	60 min	13.512±4.955	13.159±5.417	>0.9999
	120min	12.18±5.547	12.491±6.054	>0.9999
	180 min	9.163±5.139	9.073±5.090	>0.9999
Insulin &C-peptide release test				
Insulin (pmol/L)	0 min	251.95±177.864	203.276±150.821	>0.9999
	30 min	917.332±766.596	811.924±602.550	>0.9999
	60 min	1092.383±798.341	913.881±637.466	0.7816
	120min	1073.284±738.868	880.504±601.678	0.4566
	180 min	506.807±425.426	467.904±416.465	>0.9999
C-peptide (nmol/L)	0 min	1.080±0.526	0.873±0.368	>0.9999
	30 min	2.414±1.512	2.058±1.069	0.6688
	60 min	3.158±1.782	2.539±1.191	0.0534
	120min	3.008±1.474	2.702±1.044	0.7816
	180 min	1.891±0.904	1.900±.749	>0.9999
HbA1c %		7.196±1.806	6.894±1.495	0.4411
HOMA-β		118.139±59.920	118.375±71.047	0.9869
HOMA-IR		2.650±1.500	2.071±0.869	0.0643

Analyses of the bodyweight change, HbA1c, FPG, BP, plasma lipids, and lipoprotein after bariatric surgery

The findings revealed that the extent of bodyweight loss from the baseline was significant in both genders when compared to the first month with three months, six months, and one year. Similarly, blood pressure including systolic (SBP) and diastolic pressure (DBP) declined dramatically in the first month after surgery (Table 3).

**Table 3:** Comparison between male and female patients on bodyweight and blood pressure change from pre-operation. Note: pre-op: pre-operation; SBP: systolic blood pressure; DBP: diastolic blood pressure; 1 m: first month after operation; 3 m: 3 months after operation; 6 m: 6 months after operation; 1 y: 1 year after operation.

		Males	Females	P value
Bodyweight (kg)	Pre-op	121.223±20.993	97.724±19.4252	0.0016
	1 m	104.428±19.824	83.872±17.051	0.0016
	3 m	94.885±17.8302	76.221±14.827	0.0016
	6 m	88.52±15.788	71.040±12.805	0.0016
	1 y	82.93±14.154	66.87±10.778	0.0016
Blood pressure				
SBP (mmHg)	Pre-op	143.54±12.94	135.83±18.86	0.0502
	1 m	128.85±11.08	122.02±12.97	0.1170
	3 m	124.74±12.94	118.74±11.39	0.2186
	6 m	121.25±11.63	114.30±10.49	0.1626
	1 y	121.00±11.37	110.19±10.05	0.0589
DBP (mmHg)	Pre-op	90.50±10.05	85.52±11.20	0.0309
	1 m	83.11±6.53	79.14±8.69	0.1462
	3 m	82.85±7.52	76.83±6.42	0.0056
	6 m	78.67±6.23	74±5.44	0.0936
	1 y	80.47±6.41	72.50±5.70	0.0115

When comparing the amplitude of HbA1c and FPG value drop from the baseline, we found a significant decrease in both male and female patients after three months compared to those of six months and one year (Table 4).

**Table 4:** Comparison between male and female patients on the HbA1c and fasting plasma glucose (FPG) change from pre-operation. Note: HbA1c: glycated hemoglobin A1c; FPG: fasting plasma glucose; pre-op: pre-operation; 3 m: 3 months after operation; 6 m: 6 months after operation; 1 y: 1 year after operation.

		Males	Females	P value
HbA1c (%)	Pre-op	7.196±1.806	6.894±1.495	0.5988
	3 m	5.600±0.617	5.760±0.784	0.9414
	6 m	5.500±0.432	5.604±0.513	0.9914
	1 y	5.440±0.463	5.708±0.859	0.8957
FPG (mmol/L)	Pre-op	6.910±2.746	6.440±2.369	0.5776
	3 m	5.449±1.176	5.227±0.884	0.9575
	6 m	5.367±0.827	5.258±0.889	0.9980
	1 y	5.330±0.867	5.270±0.968	>0.9999

**Figure 2:**
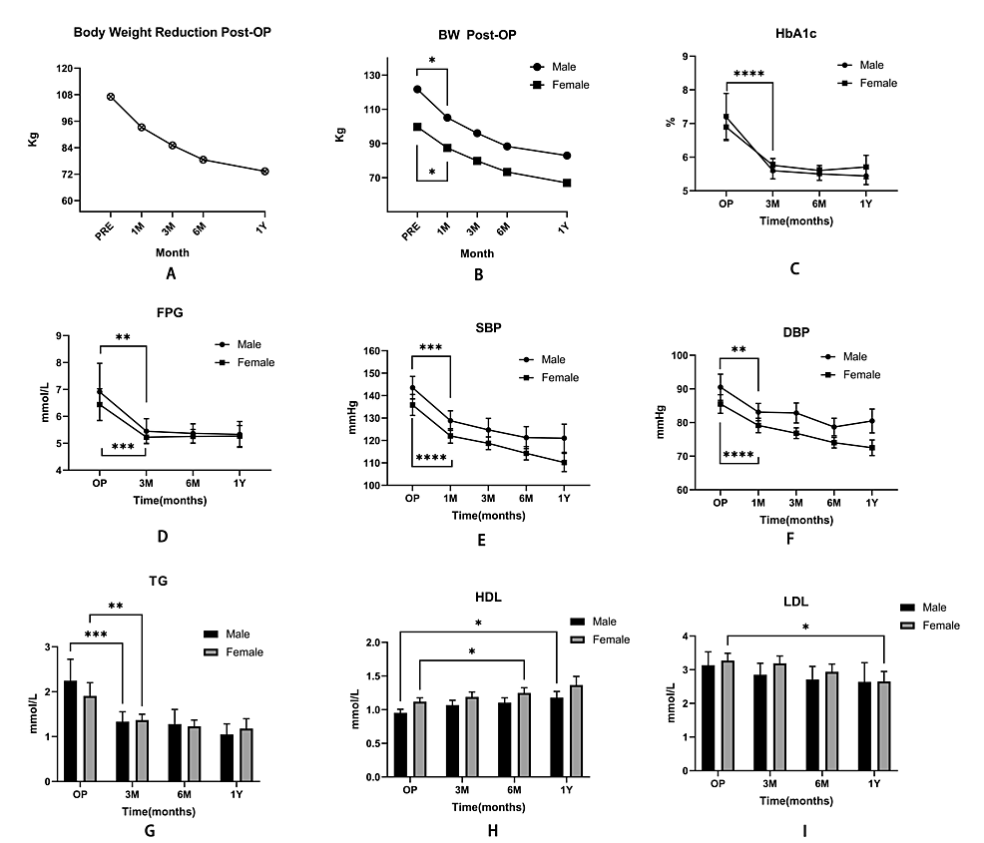
The trends on the bodyweight (A and B), HbA1c (C), fasting plasma glucose (D), systolic blood pressure (E), diastolic blood pressure (F), and plasma lipids (G-I) after surgery. Significant BMI changes occur one month after surgery, while HbA1c, FPG, and TG changes occur three months after surgery, according to the data. However, in both male and female patients, a tendency of change in HDL and LDL has lately occurred after six months. Note: *: p<0.05; **: p<0.01; ***: p<0.001; ****: p<0.0001; BW: bodyweight; PRE: the time before the surgery; 1M: one month after surgery; 3M: three months after surgery; 6M: six months after surgery; 1Y: one year after surgery; FPG: fasting plasma glucose; SBP: systolic blood pressure; DBP: diastolic blood pressure; TG: triglyceride; HDL: high-density lipoprotein; LDL: low-density lipoprotein

The TG levels, similar to HbA1c, also decreased significantly in three months. However, when compared to BMI and blood glucose, alterations in HDL and LDL were less obvious in the first one to three months after surgery. The HDL level in female patients considerably increased after six months, while male patients' HDL levels dramatically increased after a year. We only found that a significant decrease in the level of LDL occurred in females one year post-operation (Fig. 2, Table 5).

**Table 5:** Comparison between male and female patients on TG, HDL, and LDL change from pre-operation. Note: TG: triglyceride; HDL: high-density lipoprotein; LDL: low-density lipoprotein; pre-op: pre-operation; 3 m: 3 months after operation; 6 m: 6 months after operation; 1 y: 1 year after operation.

		Males	Females	P value
TG (mmol/L)	Pre-op	2.245±1.236	1.907±1.1907	0.2421
	3 m	1.335±0.554	1.369±0.516	0.9996
	6 m	1.279±0.759	1.230±0.488	0.9988
	1 y	1.048±0.425	1.179±0.542	0.9792
HDL (mmol/L)	Pre-op	0.952±0.137	1.119±0.231	0.0093
	3 m	1.068±0.179	1.190±0.288	0.1086
	6 m	1.104±0.166	1.250±0.265	0.0662
	1 y	1.179±0.166	1.366±0.316	0.0679
LDL (mmol/L)	Pre-op	3.135±1.020	3.273±0.866	0.9285
	3 m	2.850±0.866	3.193±0.862	0.3109
	6 m	2.708±0.906	2.938±0.805	0.7561
	1 y	2.637±1.032	2.652±0.732	>0.9999

Effects of independent factors on bodyweight loss and HbA1c reduction three months following bariatric surgery studied using multilinear regression

Furthermore, we established a multilinear regression model to investigate the factors that contributed to the significant change in post-operation bodyweight and HbA1c three months following bariatric surgery (Table 6). Surprisingly, the findings revealed that the bodyweight loss differentiates by gender. We discovered that, in males, pre-operation bodyweight was the only independent determinant of significant bodyweight loss in three months (β=0.798, p<0.001), whereas, in females, pre-operation bodyweight and ages were the independent determinants (pre-operation bodyweight: β=0.735, p<0.001; age: β=-0.229, p=0.004) of post-operation bodyweight. For HbA1c reduction at three months, the regression model showed that both in males and females, pre-operation HbA1c (male: β=0.935; female: β=0.919) and age (male: β=-0.181; female: β=-0.292) were the independent predictors.

**Table 6:** Multilinear regression analyses on the bodyweight and HbA1c reduction after surgery three months later in both males and females. Note: Post-BW: bodyweight; pre-BW: pre-operation bodyweight; pre-HbA1c: pre-operation HbA1c; SE: stand error; Sig: significance; VIF: variance inflation factor; R: Pearson's correlation coefficient; R2: coefficient of determination

Multilinear regression analysis		R	0.798	R^2^	0.637	Adjusted R^2^	0.623		
	Post-BW (M)	Variables	β	SE	β	t	Sig	Tolerance	VIF
		Constant	-2.792	4.351	-	-0.642	0.527	-	-
		Pre-BW	0.229	0.034	0.798	6.752	<0.001	1.000	1.000
		R	0.812	R^2^	0.660	Adjusted R^2^	0.649	-	-
	Post-BW (F)	Variables	β	SE	β	t	Sig	Tolerance	VIF
		Constant	2.996	2.366	-	1.267	0.210	-	-
		Pre-BW	0.187	0.019	0.735	9.724	<0.001	0.960	1.041
		Age	-0.096	0.032	-0.229	-3.035	0.004	0.960	1.041
		R	0.941	R^2^	0.886	Adjusted R^2^	0.877	-	-
	Post-HbA1c (M)	Variables	β	SE	β	t	Sig	Tolerance	VIF
		Constant	-3.444	0.586	-	-5.881	<0.001	-	-
		pre-HbA1c	0.855	0.062	0.935	13.840	<0.001	0.996	1.004
		Age	-0.036	0.013	-0.181	-2.676	0.013	0.996	1.004
		R	0.890	R^2^	0.792	Adjusted R^2^	0.785	-	-
	Post-HbA1c (F)	Variables	β	SE	β	t	Sig	Tolerance	VIF
		Constant	-2.810	0.334	-	-8.421	<0.001	-	-
		Pre-HbA1c	0.712	0.046	0.919	15.337	<0.001	0.934	1.071
		Age	-0.028	0.006	-0.292	-4.879	<0.001	0.934	1.071

## Discussion

Bariatric surgery was first developed to treat morbid obesity by changing the gastrointestinal tract (GI) in a variety of ways, resulting in a change in physiological digestion and absorption function, a reduction in energy intake, and rapid weight loss [8]. Bariatric surgery, on the other hand, has been demonstrated to be effective in treating hyperglycemia, dyslipidemia, hyperuricemia, hypertension, and other metabolic syndrome disorders [9-11]. Numerous clinical studies have suggested that a significant portion of the incidence of improvement in hyperglycemia or diabetes remission may be independent of bodyweight loss [12-14]. Bodyweight, FPG, HbA1c, and blood pressure all decreased rapidly in the first one to three months after surgery, according to this study. Patients' baseline conditions, such as pre-operation BMI and HbA1c, as well as their age, were the underlying clinical causes for this rapid decrease. Other factors that may have an impact after surgery include nutrition [15,16], physical activity [17], and medicines [18], which were not examined in this study. As reported in earlier research, baseline metabolic variables and age could account for 63%-88% of the causes for dramatic improvements in bodyweight and HbA1c in the first three months after surgery.

Before surgery, we compared the glycemic state and pancreatic function of the two genders. We discovered that the OGTT profile was generally similar in males and females, with a peak appearing 30 minutes to one hour after oral glucose intake and declining to fasting three hours later. In insulin and c-peptide release assays, female patients had lower insulin and c-peptide release than male patients. The difference, however, was not statistically significant. In both genders, the mean insulin and c-peptide release was less than five times that of fasting insulin and c-peptide release, indicating that pancreatic function was slightly impaired. Additionally, we noticed that HbA1c was slightly lower in female groups, indicating that female patients had better glycemic control in the three months previous to surgery than male patients. Furthermore, we utilized the HOMA model to assess pancreatic function and insulin resistance in male and female patients. While pancreatic functions were equivalent, male patients' insulin resistance was higher than female patients, which could be attributed to male patients' higher bodyweight.

Then, in both men and women, we evaluated the rate of bodyweight reduction and metabolic indicators, which were mostly consistent with previous findings [19-21]. The substantial decrease in bodyweight, HbA1c, FPG, BP, and TG levels changed in the first one to three months; however, the level of HDL and LDL changed approximately six months or one year later. We hypothesize that caloric restriction [22], relief of hyperglycemia, lipid toxicity, and insulin resistance are clinically responsible for this effect [23]. Unfortunately, we were unable to perform OGTT or insulin and c-peptide assays following surgery. Hence, we could not determine the dynamic changes in pancreatic function and insulin resistance. Nonetheless, we ultimately concluded that, in the first one to three months, a decrease in HbA1c was related to a lower mean level of FPG, indicating normalization of glucose tolerance and pancreatic function. The significantly delayed reduction in HDL and LDL may be due to the following change in bodyweight and reduced adipose tissue [24-26].

In the present study, we further explored the assessable indices in relation to the rapid loss of bodyweight and improvement of HbA1C. We discovered that pre-operation bodyweight and age were the strongest predictors of the bodyweight reduction, accounting for 63%-67% of the causes. Although it remains to be confirmed whether there is a relationship between age and bodyweight, our hypothesis regarding pre-operative bodyweight is consistent with previous evidence [27]. Because the patients were young, we believe that the gender variations in this study are due to sex hormones. Sex hormone disturbance due to metabolic syndrome before the surgery may account. The normal effect of estrogen has been associated with weight loss, adipose tissue inflammation, and insulin resistance in obese people [28]. The fact that some unpregnant female patients (n=7) were able to become pregnant following an SG in this study supports this theory.

Finally, we discovered that pre-operation HbA1c levels and age accurately predicted HbA1c change in the one to three months following surgery [29], indicating that aging is associated with a loss of pancreatic function and increased insulin resistance. The greater the HbA1c and the younger the patient, the better glycemic control or diabetes remission. The changes in gastrointestinal hormonal response [30] and gut microbiota with aging may be the additional causes for the effects we identified in this investigation.

Limitations

The conclusion was supported by research conducted at one research center. In addition, further data on alteration of the serum GLP-1, SNS activity, and adipose mass data should be included. Therefore, further research should be conducted to corroborate the recent results.

## Conclusions

Altogether, we demonstrated that, regardless of gender, bariatric surgery could effectively reduce bodyweight and contribute to the improvement of hyperglycemia, hypertension, and dyslipidemia. Age is an independent indicator that predicts the rapid change in bodyweight in females, but predicts the HbA1c changes in both genders after surgery. The different profiles of metabolic indices after bariatric surgery could be traced to the variation between genders in these young patients, according to this study.

## References

[1] Carbone S, Del Buono MG, Ozemek C, Lavie CJ (2019). Obesity, risk of diabetes and role of physical activity, exercise training and cardiorespiratory fitness. Prog Cardiovasc Dis.

[2] NCD Risk Factor Collaboration (NCD-RisC) (2016). Trends in adult body-mass index in 200 countries from 1975 to 2014: a pooled analysis of 1698 population-based measurement studies with 19·2 million participants. Lancet.

[3] Lv X, Zhou W, Sun J (2017). Visceral adiposity is significantly associated with type 2 diabetes in middle-aged and elderly Chinese women: a cross-sectional study. J Diabetes.

[4] Nguyen NT, Varela JE (2017). Bariatric surgery for obesity and metabolic disorders: state of the art. Nat Rev Gastroenterol Hepatol.

[5] Buchwald H, Buchwald JN (2019). Metabolic (bariatric and nonbariatric) surgery for type 2 diabetes: a personal perspective review. Diabetes Care.

[6] Douros JD, Tong J, D'Alessio DA (2019). The effects of bariatric surgery on islet function, insulin secretion, and glucose control. Endocr Rev.

[7] Huang R, Ding X, Fu H, Cai Q (2019). Potential mechanisms of sleeve gastrectomy for reducing weight and improving metabolism in patients with obesity. Surg Obes Relat Dis.

[8] Wolfe BM, Kvach E, Eckel RH (2016). Treatment of obesity: weight loss and bariatric surgery. Circ Res.

[9] Tsilingiris D, Koliaki C, Kokkinos A (2019). Remission of type 2 diabetes mellitus after bariatric surgery: fact or fiction?. Int J Environ Res Public Health.

[10] Hanipah ZN, Schauer PR (2020). Bariatric surgery as a long-term treatment for type 2 diabetes/metabolic syndrome. Annu Rev Med.

[11] Barron M, Atkinson SN, Kirby J, Kindel T (2021). Sleeve gastrectomy prevents hypertension associated with unique shifts in the gut microbiome. Surg Endosc.

[12] Mingrone G, Panunzi S, De Gaetano A (2021). Metabolic surgery versus conventional medical therapy in patients with type 2 diabetes: 10-year follow-up of an open-label, single-centre, randomised controlled trial. Lancet.

[13] Kong X, Tu Y, Li B (2019). Roux-en-Y gastric bypass enhances insulin secretion in type 2 diabetes via FXR-mediated TRPA1 expression. Mol Metab.

[14] Simonson DC, Vernon A, Foster K, Halperin F, Patti ME, Goldfine AB (2019). Adjustable gastric band surgery or medical management in patients with type 2 diabetes and obesity: three-year results of a randomized trial. Surg Obes Relat Dis.

[15] Uribarri-Gonzalez L, Nieto-García L, Martis-Sueiro A, Dominguez-Muñoz JE (2021). Exocrine pancreatic function and dynamic of digestion after restrictive and malabsorptive bariatric surgery: a prospective, cross-sectional, and comparative study. Surg Obes Relat Dis.

[16] Wang Y, Duan L, Han X, Wang J, Yan G (2022). Changes in nutritional outcomes after sleeve gastrectomy: a systematic review and meta-analysis. Obes Surg.

[17] Gil S, Kirwan JP, Murai IH (2021). A randomized clinical trial on the effects of exercise on muscle remodelling following bariatric surgery. J Cachexia Sarcopenia Muscle.

[18] Kingma JS, Burgers DM, Monpellier VM (2021). Oral drug dosing following bariatric surgery: general concepts and specific dosing advice. Br J Clin Pharmacol.

[19] Singh D, Baksi A, Ramana P, Singla V, Aggarwal S (2022). Five-year outcomes of sleeve gastrectomy in patients with class I obesity and type 2 diabetes mellitus. Obes Surg.

[20] Nuijten MA, Eijsvogels TM, Monpellier VM, Janssen IM, Hazebroek EJ, Hopman MT (2022). The magnitude and progress of lean body mass, fat-free mass, and skeletal muscle mass loss following bariatric surgery: a systematic review and meta-analysis. Obes Rev.

[21] Gomes-Rocha SR, Costa-Pinho AM, Pais-Neto CC (2022). Roux-en-Y gastric bypass vs sleeve gastrectomy in super obesity: a systematic review and meta-analysis. Obes Surg.

[22] Herzog K, Berggren J, Al Majdoub M (2020). Metabolic effects of gastric bypass surgery: is it all about calories?. Diabetes.

[23] Shi Q, Wang Q, Zhong H (2021). Roux-en-Y gastric bypass improved insulin resistance via alteration of the Human Gut microbiome and alleviation of endotoxemia. Biomed Res Int.

[24] Lee JK, Park YS, Kim K, Oh TJ, Chang W (2021). Comparison of bioelectrical impedance analysis and computed tomography on body composition changes including visceral fat after bariatric surgery in Asian patients with obesity. Obes Surg.

[25] Carruthers NJ, Strieder-Barboza C, Caruso JA (2021). The human type 2 diabetes-specific visceral adipose tissue proteome and transcriptome in obesity. Sci Rep.

[26] Bettini S, Segato G, Prevedello L (2021). Improvement of lipid profile after one-anastomosis gastric bypass compared to sleeve gastrectomy. Nutrients.

[27] Schauer PR, Bhatt DL, Kirwan JP (2017). Bariatric surgery versus intensive medical therapy for diabetes - 5-year outcomes. N Engl J Med.

[28] Różańska-Walędziak A, Bartnik P, Kacperczyk-Bartnik J, Czajkowski K, Walędziak M (2020). The impact of bariatric surgery on menstrual abnormalities-a cross-sectional study. Obes Surg.

[29] Nasta AM, Goel R, Goel M, Malek A (2021). Undiagnosed hyperglycemia is a potential long-term risk in metabolic surgery patients: 7 years follow-up study. Obes Surg.

[30] Jones B, Sands C, Alexiadou K (2022). The metabolomic effects of tripeptide gut hormone infusion compared to Roux-en-Y gastric bypass and caloric restriction. J Clin Endocrinol Metab.

